# A Multicenter Comparative Study of Neurosurgical and Orthopedic Spine Services for the Management of Spinal Conditions at Level I Trauma Centers

**DOI:** 10.7759/cureus.90648

**Published:** 2025-08-21

**Authors:** Ehtisham Ahmed, Asghar Ali, Muhammad Tayyab, Muhammad Tanveer, Ujala Gulzaib, Haider Ali Khattak, Usama Mansoor

**Affiliations:** 1 Neurosurgery, Ayub Medical College, Abbottabad, PAK; 2 Neurosurgery, Bacha Khan Medical College, Mardan, PAK; 3 Trauma and Orthopaedics, Bradford Teaching Hospitals National Health Service (NHS) Foundation Trust, Bradford, GBR; 4 Trauma and Orthopaedics, University Hospitals of North Midlands National Health Service (NHS) Trust, Stoke-on-Trent, GBR; 5 Biomedical Engineering and Sciences (BMES), School of Mechanical and Manufacturing Engineering (SMME) National University of Sciences and Technology, Islamabad, PAK; 6 Neurosurgery, Punjab Institute of Neurosciences, Lahore, PAK

**Keywords:** multidisciplinary care, neurosurgery, orthopedic spine surgery, spinal trauma, surgical outcomes, trauma centers

## Abstract

Background: Spinal conditions are commonly managed by both neurosurgeons and orthopedic spine surgeons, but differences in clinical approaches and outcomes remain underexplored.

Objective: The objective of the study is to compare patient demographics, consultation patterns, surgical techniques, and early postoperative outcomes between neurosurgery and orthopedic spine services at Level I trauma centers.

Methodology: This retrospective multicenter study analyzed 690 patients who presented with spinal conditions and were treated either by neurosurgery (n = 355; 51.5%) or orthopedic (n = 335; 48.5%) spine services. Variables assessed included age, gender, injury type, spinal region involved, imaging utilization, management type, surgical techniques, operative duration, hospital stay, complications, ICU admissions, and discharge status. Statistical analysis was performed using SPSS version 26.0 (IBM Corp., Armonk, NY, US). Chi-squared and independent t-tests compared categorical and continuous variables, respectively, with significance set at p < 0.05.

Results: Mean patient age was comparable between groups (47.25 ± 16.4 years in neurosurgery vs. 45.80 ± 15.9 years in orthopedics, p = 0.231). Male patients comprised 238 (67.04%) in neurosurgery and 223 (66.57%) in orthopedics (p = 0.911). Traumatic injuries accounted for 203 (57.18%) neurosurgery and 188 (56.12%) orthopedic cases (p = 0.782). Lumbar spine involvement was higher in orthopedic patients with 151 (45.07%) vs. 132 (37.18%) in neurosurgery (p = 0.042). Operative management was performed in 195 (54.93%) neurosurgical and 181 (54.03%) orthopedic patients (p = 0.811). Neurosurgeons more frequently performed decompression with fusion (132, 67.69% vs. 101, 55.80%, p = 0.017) and used anterior or combined approaches (106, 54.36% vs. 70, 38.67%, p = 0.002), with longer mean operative times (152.6 ± 37.5 minutes vs. 138.9 ± 34.2 minutes, p = 0.003). Postoperative complications occurred in 41 (11.55%) neurosurgical and 36 (10.75%) orthopedic patients (p = 0.735). ICU admissions were similar between the two groups, with 54 patients (15.21%) in the neurosurgery group and 49 patients (14.63%) in the orthopedic group (p = 0.847). Likewise, 30-day readmission rates did not differ significantly, with 12 patients (3.38%) readmitted in the neurosurgery group compared to nine patients (2.69%) in the orthopedic group (p = 0.579).

Conclusion: Despite differences in surgical approaches, neurosurgery and orthopedic spine services demonstrated comparable consultation patterns and clinical outcomes, supporting multidisciplinary collaboration in trauma care.

## Introduction

A large percentage of the world's illness burden is caused by spinal injuries and abnormalities, which often require specialized treatment [1,2]. Patients with spinal disorders are often referred to either orthopedic spine services or neurosurgery at tertiary care facilities [3]. The clinical approach, diagnostic assessment, surgical decision-making, and long-term treatment methods of the two specialties may vary, even if they share capabilities in treating spinal disorders [4]. Even if they are minor, these differences may have an impact on patient outcomes, resource use, and the effectiveness of healthcare as a whole [5].

Level I trauma centers are perfect places to assess and contrast subspecialty practices since they often operate as the main hubs for treating high-acuity spine injuries [6,7]. Both orthopedic spine surgeons and neurosurgeons often assess trauma patients in these settings, sometimes working together and other times working alone [8]. Nonetheless, disparities in the timing of surgical intervention, the choice of surgical technique, consultation patterns, and postoperative care procedures are often seen [9]. Standardizing treatment pathways and maximizing multidisciplinary cooperation need an understanding of how these variations appear in clinical practice [10].

Prior research, which mostly focused on elective patients, examined the results of spine procedures carried out by neurosurgeons as opposed to orthopedic surgeons [11]. However, there is still a dearth of research on variations in spine consults associated with trauma, especially in high-volume trauma centers. Because spine trauma is complicated and often urgent, the specialty offering the first consultation may have an impact on patient recovery, intervention decisions, and care schedules.

Furthermore, consultation patterns and treatment results may be impacted by department-specific procedures, surgeon competence, and institutional rules. This emphasizes how important it is to thoroughly assess actual consultation procedures and their effects on trauma treatment. Comparative studies may provide light on possible duplications, hold-ups, or benefits of including either specialty.

Research objective

The research objective is to compare consultation trends, clinical decision-making patterns, and treatment approaches between neurosurgery and orthopedic spine services in the management of spinal conditions at a single Level I trauma center.

## Materials and methods

Study design and setting

This retrospective, observational, multicenter study was conducted at Mardan Medical Complex, Mardan, KPK, Pakistan, and Ayub Medical College, Abbottabad, Pakistan. Both institutions are designated Level I trauma centers and receive a high volume of spinal consultations, making them suitable for evaluating specialty-specific consultation trends and clinical practices. The study covered a two-year period from July 2022 to June 2024.

Inclusion and exclusion criteria

During the research period, patients who were 18 years of age or older and had a spinal consultation for either traumatic or non-traumatic spine disorders from the neurosurgical or orthopedic spine service were eligible to be included. Incomplete or missing medical records, transfers to another institution prior to the start of treatment, duplicate entries in the record system, and patients under the age of 18 were all grounds for exclusion.

Sampling and sample size

A total of 690 patients were enrolled using convenience sampling from the eligible inpatient and emergency spine consultation population across both study centers. The rationale for using convenience sampling was the observational, multicenter design and the intent to include all consecutive patients meeting the inclusion criteria during the study duration. A formal a priori power or sample size calculation was not conducted, as the study was exploratory in nature and designed to reflect real-world clinical practice differences between the two specialties. Nonetheless, the final sample size of 690 is larger than a prior observational study comparing neurosurgical and orthopedic spine practices, lending credibility to the robustness of the analysis [12]. This limitation has been acknowledged in the discussion.

Group allocation

The neurosurgery team (n = 355) and the orthopedic spine team (n = 335) were the consulting specialties that treated the patients' spinal conditions. Clinical triage methods, standard hospital procedures, and the availability of consultants at the time of patient presentation all played a role in group assignment. The two specialties’ consultation patterns, treatment approaches, and patient results may be objectively compared because of this natural divide, which mirrors actual clinical practice.

Data collection

The study team worked with neurosurgery and orthopedic physicians to design a standardized data collection form, which was used to gather data retrospectively from hospital records and computerized systems at both centers. Data extraction was done by trained medical officers and research assistants who were given advanced training to guarantee uniformity. Patient demographics, the type of spinal condition, the specialty consulted, the time and type of the consultation, diagnostic assessments, treatment suggestions, operative versus non-operative management, surgical specifics if relevant, and patient outcomes like hospital stay duration, complication rates, and discharge status were among the important data points.

Outcomes

In addition to hospital-based outcomes, the primary focus of this study was the assessment of preoperative and postoperative neurological status. Four core domains were evaluated: (1) motor power, (2) muscle tone, (3) sensory function, and (4) bowel and bladder involvement. Motor power was assessed using the Medical Research Council (MRC) scale; tone was classified as normal, spastic, or flaccid; sensation was documented as intact, diminished, or absent; and bowel/bladder function was categorized as normal, urgency/incontinence, or retention requiring catheterization.

Additional pre- and postoperative variables included deep tendon reflexes (present, diminished, and absent), pathological reflexes (Babinski, clonus), pain severity using the Visual Analog Scale (VAS), and functional mobility (independent, assisted, or wheelchair-bound). Radiological stability (preoperative instability vs. postoperative stabilization/fusion) was noted where imaging follow-up was available.

Neurological and functional status was assessed at baseline (pre-op or at admission for non-operative cases) and at discharge (post-op or at the end of admission). Outcomes were categorized as improved, unchanged, or worsened in each domain.

Statistical analysis

SPSS version 26.0 (IBM Corp., Armonk, NY, US) was used to conduct the statistical analysis. Clinical features and patient demographics were compiled using descriptive statistics. Continuous data were shown as means with standard deviations, while categorical variables were shown as frequencies and percentages. Continuous variables were compared using the independent t-test, while categorical variables were compared across groups using the chi-squared test. Preoperative and postoperative neurological outcomes (power, tone, sensation, reflexes, bowel/bladder function, pain, and mobility) were compared using paired analyses within each specialty, and chi-squared/t-tests between specialties, with significance set at p < 0.05.

Ethical approval

The Institutional Review Boards (IRBs) examined and approved the research protocol. The Declaration of Helsinki's ethical guidelines were followed while conducting the research. To ensure confidentiality during the study procedure, all patient data were anonymized.

## Results

Of the 690 patients, 335 (48.55%) were treated by orthopedic spine services and 355 (51.5%) by neurosurgery (Table 1). For neurosurgery, the mean age was 47.25 ± 16.4 years, whereas for orthopedics, it was 45.80 ± 15.9 years (p = 0.231); 67.04% (n = 238) of the neurosurgery group and 66.57% (n = 223) of the orthopedic group were male patients, while 32.96% (n = 117) and 33.43% (n = 112) of the patients were female (p = 0.911). There was no significant difference (p = 0.765) in the prevalence of urban living between the two groups, with 198 patients (55.77%) in neurosurgery and 183 patients (54.63%) in orthopedics.

**Table 1:** Baseline Demographics of Patients by Consulting Specialty SD: standard deviation

Variable category	Variable	Neurosurgery (n = 355)	Orthopedic spine (n = 335)	p-value (test used)	Test statistic (t/χ²)
Demographic variables	Mean age (years ± SD)	47.25 ± 16.4	45.80 ± 15.9	0.231 (independent t-test)	t = 1.2
	Male (n; %)	238 (67.04%)	223 (66.57%)	0.911 (chi-squared test)	χ² = 0.012
	Female (n; %)	117 (32.96%)	112 (33.43%)	-	-
Residential status	Urban residence (n; %)	198 (55.77%)	183 (54.63%)	0.765 (chi-squared test)	χ² = 0.090
	Rural residence (n; %)	157 (44.23%)	152 (45.37%)	-	-

Out of the 690 patients, 203 neurosurgery cases (57.18%) and 188 orthopedic cases (56.12%) had traumatic spinal injuries as the major cause, while 152 (42.82%) and 147 (43.88%) cases had non-traumatic conditions (p = 0.782) (Table 2). Regarding spinal region involvement, 132 neurosurgical patients (37.18%) and 108 orthopedic patients (32.24%) had cervical spine problems (p = 0.174), 91 (25.63%) and 76 (22.69%) had thoracic involvement (p = 0.398), and 151 patients (45.07%) in the orthopedic group had lumbar spine pathology, which was statistically significant (p = 0.042) compared to 132 neurosurgical patients (37.18%).

**Table 2:** Nature and Type of Spinal Condition by Consulting Specialty

Variable category	Variable	Neurosurgery (n = 355)	Orthopedic spine (n = 335)	p-value (test used)	Test statistic (χ²)
Etiology of condition	Traumatic injury (n; %)	203 (57.18%)	188 (56.12%)	0.782 (chi-squared test)	χ² = 0.077
	Non-traumatic pathology (n; %)	152 (42.82%)	147 (43.88%)	-	-
Spinal region involved	Cervical spine (n; %)	132 (37.18%)	108 (32.24%)	0.174 (chi-squared test)	χ² = 1.85
	Thoracic spine (n; %)	91 (25.63%)	76 (22.69%)	0.398 (chi-squared test)	χ² = 0.71
	Lumbar spine (n; %)	132 (37.18%)	151 (45.07%)	0.042 (chi-squared test)	χ² = 4.14

Early consultation (within six hours) occurred in 267 neurosurgical cases (75.21%) versus 239 orthopedic cases (71.34%) (p = 0.276), shown in Table 3. MRI was used in 301 neurosurgery patients (84.79%) and 274 orthopedic cases (81.79%) (p = 0.318), while CT scans were performed in 281 (79.15%) and 263 (78.51%), respectively (p = 0.846). Operative management was nearly identical between groups: 195 neurosurgery patients (54.93%) and 181 orthopedic patients (54.03%) underwent surgery, while non-operative care was provided to 160 (45.07%) and 154 (45.97%) patients, respectively (p = 0.811).

**Table 3:** Consultation and Management Patterns by Consulting Specialty

Variable category	Variable	Neurosurgery (n = 355)	Orthopedic spine (n = 335)	p-value (test used)	Test statistic (χ²)
Consultation timing	Consult within 6 hrs (n; %)	267 (75.21%)	239 (71.34%)	0.276 (chi-squared test)	χ² = 1.19
Imaging utilization	MRI utilized (n; %)	301 (84.79%)	274 (81.79%)	0.318 (chi-squared test)	χ² = 1.00
	CT scan performed (n; %)	281 (79.15%)	263 (78.51%)	0.846 (chi-squared test)	χ² = 0.038
Management approach	Operative management (n; %)	195 (54.93%)	181 (54.03%)	0.811 (chi-squared test)	χ² = 0.058
	Non-operative management (n; %)	160 (45.07%)	154 (45.97%)	-	-

Among 376 operated patients (195 neurosurgeries and 181 orthopedics), decompression with fusion was more frequently performed by neurosurgeons (n = 132; 67.69%) compared to orthopedic surgeons (n = 101; 55.80%) (p = 0.017), shown in Table 4. Orthopedic surgeons favored the posterior-only approach (n = 111; 61.33%) more than neurosurgeons (n = 89; 45.64%) (p = 0.002), while neurosurgeons used anterior or combined approaches more often (n = 106; 54.36% vs. n = 70; 38.67%). Neurosurgical procedures also had a significantly longer mean duration of 152.6 ± 37.5 minutes compared to 138.9 ± 34.2 minutes for orthopedics (p = 0.003).

**Table 4:** Surgical Details (Operated Cases Only) SD: standard deviation

Variable category	Variable	Neurosurgery (n = 195)	Orthopedic spine (n = 181)	p-value (test used)	Test statistic (t/χ²)
Surgical technique	Decompression with fusion (n; %)	132 (67.69%)	101 (55.80%)	0.017 (chi-squared test)	χ² = 5.72
	Posterior-only approach (n; %)	89 (45.64%)	111 (61.33%)	0.002 (chi-squared test)	χ² = 9.47
	Anterior or combined (n; %)	106 (54.36%)	70 (38.67%)	-	-
Surgical characteristics	Avg. surgery duration (min ± SD)	152.6 ± 37.5	138.9 ± 34.2	0.003 (independent t-test)	t = 2.97

Neurological recovery was broadly comparable between neurosurgery and orthopedic spine services. Motor power improved in 52.4% of neurosurgery patients vs. 51.0% of orthopedic patients (odds ratio (OR) 1.05, 95% CI 0.78-1.41; p = 0.721), while sensory recovery was noted in 37.5% vs. 33.4% (OR 1.19, 95% CI 0.86-1.64; p = 0.287), respectively. Bowel and bladder function improved in 27.6% vs. 25.1% (OR 1.14, 95% CI 0.80-1.62; p = 0.473). Pain reduction of at least two VAS points occurred in 56.6% vs. 54.3% (OR 1.10, 95% CI 0.82-1.48; p = 0.544), while functional mobility gains were seen in 49.0% vs. 47.5% (OR 1.07, 95% CI 0.80-1.44; p = 0.689) of patients. None of these neurological outcomes reached statistical significance, indicating similar recovery profiles across specialties.

Hospital course metrics also showed minimal differences between the two groups. The average length of stay was 6.7 ± 3.1 days for neurosurgery vs. 6.2 ± 2.9 days for orthopedics (MD 0.48 days, 95% CI 0.02-0.94; p = 0.038), representing the only statistically significant difference. Postoperative complications occurred in 11.6% vs. 10.8% (p = 0.735), ICU admission in 15.2% vs. 14.6% (p = 0.847), 30-day readmission in 3.4% vs. 2.7% (p = 0.579), and discharge to rehabilitation in 25.1% vs. 22.7% (p = 0.481) of patients, with no significant inter-specialty variation. Table 5 provides a detailed summary of both neurological recovery and hospital-based outcomes across the two specialties.

**Table 5:** Postoperative and Clinical Outcomes MRC: Medical Research Council; VAS: Visual Analog Scale; SD: standard deviation; MD: mean difference

Outcome variable		Neurosurgery (n = 355)	Orthopedic spine (n = 335)	p-value	Test used	Test statistic	Odds ratio (OR) (95% CI)
Primary outcomes: neurological recovery	Motor power improvement (≥1 MRC grade)	186 (52.39%)	171 (51.04%)	0.721	Chi square	χ² = 0.13	OR = 1.05 (0.78–1.41)
	Muscle tone normalized	142 (40.00%)	128 (38.21%)	0.647	Chi square	χ² = 0.21	OR = 1.08 (0.79–1.47)
	Sensory function improved	133 (37.46%)	112 (33.43%)	0.287	Chi square	χ² = 1.13	OR = 1.19 (0.86–1.64)
	Bowel & bladder function improved	98 (27.61%)	84 (25.07%)	0.473	Chi square	χ² = 0.52	OR = 1.14 (0.80–1.62)
	Reflexes normalized	121 (34.08%)	109 (32.54%)	0.679	Chi square	χ² = 0.17	OR = 1.07 (0.77–1.49)
	Pain reduction (VAS ≥ 2 points)	201 (56.62%)	182 (54.33%)	0.544	Chi square	χ² = 0.37	OR = 1.10 (0.82–1.48)
	Functional mobility improved	174 (49.01%)	159 (47.46%)	0.689	Chi square	χ² = 0.16	OR = 1.07 (0.80–1.44)
	Mean pain score change (VAS, mean ± SD)	-2.8 ± 1.6	-2.6 ± 1.5	0.212	Independent t-test	t = 1.25	MD = -0.2 (95% CI -0.5–0.1)
Secondary outcomes–hospital course	Avg. length of stay (days ± SD)	6.72 ± 3.1	6.24 ± 2.9	0.038	Independent t-test	t = 2.08	MD = 0.48 (95% CI 0.02–0.94)
	Postoperative complications	41 (11.55%)	36 (10.75%)	0.735	Chi square	χ² = 0.114	OR = 1.08 (0.67–1.73)
	ICU admission	54 (15.21%)	49 (14.63%)	0.847	Chi square	χ² = 0.038	OR = 1.05 (0.69–1.59)
	30-day readmission	12 (3.38%)	9 (2.69%)	0.579	Chi square	χ² = 0.31	OR = 1.26 (0.54–2.94)
	Discharge to rehabilitation	89 (25.07%)	76 (22.69%)	0.481	Chi square	χ² = 0.50	OR = 1.14 (0.80–1.63)

## Discussion

The present multicenter study compared neurosurgical and orthopedic spine services in the management of spinal conditions at Level I trauma centers, focusing on consultation trends, decision-making patterns, surgical approaches, and outcomes. Our analysis revealed several notable differences and similarities that both confirm and expand upon existing literature.

Demographically, there was no statistically significant difference between the two groups regarding age (47.25 ± 16.4 years vs. 45.80 ± 15.9 years, p = 0.231) or gender distribution (male: 67.04% vs. 66.57%, p = 0.911). These findings are consistent with the previous research study, which reported comparable baseline characteristics across specialties in spinal trauma cases [13]. The near-equal distribution supports the robustness of our comparative outcomes.

In terms of spinal pathology, both specialties managed a similar proportion of traumatic cases-57.18% in neurosurgery versus 56.12% in orthopedics (p = 0.782). However, lumbar spine involvement was significantly more common in orthopedic cases (45.07% vs. 37.18%, p = 0.042), which may reflect training emphasis or subspecialty preference. This observation aligns with a previous study, where orthopedic surgeons were more frequently involved in lumbar pathologies compared to cervical or thoracic cases [14].

Regarding consultation and management trends, early consultation (within six hours) occurred in 75.21% of neurosurgical cases and 71.34% of orthopedic cases (p = 0.276), indicating similar responsiveness. The use of MRI and CT imaging was also comparable between specialties. However, operative intervention rates were nearly identical (neurosurgery: 54.93%, orthopedics: 54.03%, p = 0.811), reinforcing findings from a previous study that showed no significant difference in operative decision-making across disciplines when adjusted for injury severity [15,16].

Surgical techniques did differ significantly. Neurosurgeons performed decompression with fusion more often (67.69% vs. 55.80%, p = 0.017), while orthopedic surgeons favored posterior-only approaches (61.33% vs. 45.64%, p = 0.002). Similar patterns were reported in a previous study, suggesting neurosurgeons are more likely to employ anterior or combined approaches, possibly due to comfort with ventral spinal anatomy [17]. Moreover, the longer mean operative time for neurosurgery (152.6 ± 37.5 minutes vs. 138.9 ± 34.2 minutes, p = 0.003) may reflect the complexity of these approaches.

Postoperative outcomes, including complication rates (11.55% vs. 10.75%, p = 0.735) and ICU admissions (15.21% vs. 14.63%, p = 0.847), were statistically comparable, supporting previous findings that both specialties achieve similar complication profiles when matched for case severity [18]. However, neurosurgery patients had a slightly longer hospital stay (6.72 ± 3.1 vs. 6.24 ± 2.9 days, p = 0.038), which could be linked to longer procedures or greater use of complex approaches.

Strengths and limitations

This multicenter study offers valuable insights into the comparative clinical practices of neurosurgery and orthopedic spine services in managing spinal conditions at Level I trauma centers. Its key strengths include a robust sample size (n = 690), standardized data collection, and real-world applicability due to unbiased patient allocation based on routine triage protocols. The comparative design enhances our understanding of specialty-specific decision-making patterns and outcomes. However, the retrospective nature of the study introduces potential limitations such as documentation bias and lack of control over confounding variables. Additionally, the absence of long-term functional outcomes, limited geographic scope to two centers, and unstratified analysis of injury severity (e.g., American Spinal Injury Association (ASIA) grades or specific fracture classifications) may restrict the generalizability and depth of the findings.

## Conclusions

In treating spinal diseases in trauma centers, this research emphasizes the unique and overlapping functions of neurosurgery and orthopedic spine services. Significant variations were seen in the chosen surgical techniques and operating times, despite the fact that both specialties showed comparable consultation patterns, operative rates, and complication profiles. Orthopedic surgeons more often used posterior-only methods, while neurosurgeons preferred decompression with fusion and mixed treatments. Patient outcomes, including ICU admissions, surgical complications, and discharge disposition, were generally similar in spite of these differences. Neurological recovery outcomes (motor power, tone, sensation, bowel/bladder, reflexes, pain, and mobility) were broadly comparable between the two specialties, with the only significant difference being a slightly longer hospital stay among neurosurgical patients. In order to maximize results and resource utilization in trauma settings, our findings highlight the need for multidisciplinary teamwork and support the inclusion of both specialties in standardized spine care pathways.
